# Exploratory Efficacy of Calcium-Vitamin D Milk Fortification and Periodontal Therapy on Maternal Oral Health and Metabolic and Inflammatory Profile

**DOI:** 10.3390/nu13030783

**Published:** 2021-02-27

**Authors:** Amanda Rodrigues Amorim Adegboye, Danilo Dias Santana, Pedro Paulo Teixeira dos Santos, Paula Guedes Cocate, Camila Benaim, Maria Beatriz Trindade de Castro, Michael Maia Schlüssel, Gilberto Kac, Berit Lilienthal Heitmann

**Affiliations:** 1 Faculty of Health and Life Sciences, School of Nursing, Midwifery and Health, Coventry University, Priory Street, Coventry CV1 5FB, UK; 2 Nutritional Epidemiology Observatory, Department of Social and Applied Nutrition, Institute of Nutrition Josué de Castro, Federal University of Rio de Janeiro, Rio de Janeiro 21941-902, Brazil; dias.danilo@hotmail.com (D.D.S.); dr2p@hotmail.com (P.P.T.d.S.); camilabenaimnutri@gmail.com (C.B.); mbtcastro@gmail.com (M.B.T.d.C.); gilberto.kac@gmail.com (G.K.); 3 Department of Bioscience and Physical Activity, School of Physical Education and Sports, Federal University of Rio de Janeiro, Rio de Janeiro 21941-599, Brazil; paulacocate@gmail.com; 4 The EQUATOR Network—UK Centre, Centre for Statistics in Medicine, Nuffield Department of Orthopaedics, Rheumatology and Musculoskeletal Sciences, University of Oxford, Old Road, Oxford OX3 7LD, UK; michael.schlussel@csm.ox.ac.uk; 5 Research Unit for Dietary Studies at the Parker Institute, Bispebjerg and Frederiksberg Hospital, The Capital Region, 2000 Frederiksberg, Denmark; Berit.Lilienthal.Heitmann@regionh.dk; 6 Section for General Practice, Institute of Public Health, Copenhagen University, 1014 Copenhagen, Denmark

**Keywords:** exploratory efficacy, feasibility trial, pregnant women, calcium, vitamin D, periodontitis

## Abstract

In this 2 × 2 factorial, outcome-assessor blinded, feasibility randomised trial we explored the effect of a non-pharmaceutical multi-component intervention on periodontal health and metabolic and inflammatory profiles among pregnant women with periodontitis receiving prenatal care in a Brazilian public health centre. 69 pregnant women (gestational age ≤20 weeks, T0) were randomly allocated into four groups: (1) fortified sachet (vitamin D and calcium) and powdered milk plus periodontal therapy during pregnancy (early PT) (*n* = 17); (2) placebo sachet and powdered milk plus early PT (*n* = 15); (3) fortified sachet and powdered milk plus late PT (after delivery) (*n* = 19); (4) placebo sachet and powdered milk plus late PT (*n* = 18). Third trimester (T1) and 6–8 weeks postpartum (T2) exploratory outcomes included periodontal health (% sites with bleeding on probing (BOP)), glucose, insulin, C-Reactive Protein, serum calcium and vitamin D. The mean BOP was significantly reduced in the early PT groups, while BOP worsened in the late PT groups. No significant effect of fortification on BOP was observed. Changes in glucose levels and variation on birthweight did not differ among groups This feasibility trial provides preliminary evidence for estimating the minimum clinically important differences for selected maternal outcomes. A large-scale trial to evaluate the interventions’ clinical benefits and cost-effectiveness is warranted.

## 1. Introduction

Periodontitis is a chronic inflammatory disease affecting the periodontium and tooth-supporting structures such as alveolar bone and its progression can lead to tooth loss [1]. Periodontitis is a highly prevalent condition among adults and affects 10–43.7% of the general adult population worldwide [2]. While periodontitis is common in women of reproductive age, gingival inflammation tends to worsen during pregnancy due to hormonal changes [3,4,5]. This inflammatory burden can affect maternal health beyond the oral cavity leading to a low-grade systemic inflammatory status and metabolic disturbances, influencing the course of gestation [6]. There is evidence supporting the association between periodontal inflammation and diabetes [7]. Treating or reducing inflammatory periodontal diseases results in improvements in glycaemic control which in turn can reduce microvascular complications of diabetes [7].

Maternal chronic periodontitis thus increases the risk of poor pregnancy and birth outcomes including preterm delivery, low birth weight, gestational diabetes and pre-eclampsia [6,8,9]. Fortunately, periodontitis is a treatable and preventable disease and screening for this condition during pregnancy should not be overlooked by health care professionals. However, previous systematic reviews on the effect of periodontal treatment during pregnancy on maternal inflammatory and metabolic profile and gestational outcomes yielded inconsistent results [10,11,12].

A systematic review with meta-analysis including four randomised clinical trials (RCT) of non-surgical periodontal treatment during pregnancy failed to demonstrate consistent improvement in gestational outcomes [11]. For instance, the review found an overall reduction of 46% in the risk of preterm delivery, but no significant reduction in the risk for low birth weight [11]. Although periodontal therapy improved maternal periodontal clinical parameters, no significant reduction in the levels of inflammatory markers in cord blood samples was detected [11]. Another systematic review including twenty RCTs investigated the effect of any type of periodontal treatment during pregnancy on perinatal mortality and found a significantly decreased risk of perinatal mortality among those in the treatment group [10]. The review also found a reduction in preterm birth and an increase in birthweight. However, periodontal treatment did not reduce the risk of preeclampsia, gestational diabetes, cesarean section, small for gestational age, or congenital malformations [10]. Finally, a recent systematic review, including 19 RCTs on non-surgical periodontal treatment during pregnancy [12], concluded that this type of therapy is safe during pregnancy, and even though it does not entirely prevent adverse pregnancy outcomes, it can be safely recommended as a part of routine prenatal care. Considering the available evidence, it seems desirable to find co-adjuvant therapies that could enhance the benefits of non-surgical periodontal treatment during pregnancy.

Periodontitis is a multifactorial condition and there is evidence that diet and nutritional status play an important role in the disease progression [13]. Observational studies have shown that vitamin D and calcium deficiency result in increased inflammation and bone loss, suggesting that low vitamin D and calcium levels are risk factors for periodontitis [14] and that adequate intakes of vitamin D and calcium, particularly from milk, are associated with a lower risk of dental plaque [15], periodontitis [14,16,17] and tooth loss [18] in adults. A cross-sectional study in an adult population evaluated the serum levels of vitamin D and calcium in periodontally healthy, chronic gingivitis and chronic periodontitis patients with and without type two diabetes mellitus [19]. The study consistently found that vitamin D and calcium levels were inversely correlated with random blood sugar and glycated haemoglobin and also pocket depth and clinical attachment loss, thus contributing towards an increase in periodontal disease severity [19].

A recent meta-analysis found that calcium and vitamin D supplementation significantly improved clinical responses to non-surgical periodontal treatment compared to placebo [20]. However, no previous studies have been conducted among pregnant women.

Furthermore, dietary supplementation with vitamin D and calcium has other health benefits beyond those for oral health and a recent RCT found that calcium-vitamin D co-supplementation for nine weeks during pregnancy among women at high-risk for pre-eclampsia resulted in lower fast plasma glucose and serum triglycerides levels in the intervention group compared to control [21]. It has also been observed that calcium-vitamin D co-supplementation significantly decreased C-reactive protein and blood pressure [22]. Yet, both the intakes of dairy and calcium [23] and access to periodontal therapy are disproportionally low in South America and other low- and middle-income countries compared to high-income countries.

We, therefore, undertook a small-scale trial on non-surgical periodontal treatment combined with milk fortification with vitamin D and calcium during pregnancy in a deprived area of Rio de Janeiro to investigate its feasibility [24,25]. Exploratory studies, also known as pilot and feasibility studies, are crucial in assessing the feasibility and value of progressing to an effectiveness study, and by this means reducing costs and minimising potential harms of interventions [26]. Additionally, exploratory studies provide a preliminary assessment of effect sizes which can inform an accurate estimation of the sample size for the full-scale trial and other trial design parameters [26]. We found that our multi-component intervention was well tolerated and accepted by pregnant women and that the trial design was feasible with minor modifications [24]. In this paper, the potential impact of this exploratory feasibility trial on maternal periodontal clinical outcomes, inflammatory and metabolic markers are reported.

## 2. Methods

### 2.1. Study Design

**Figure 1:**
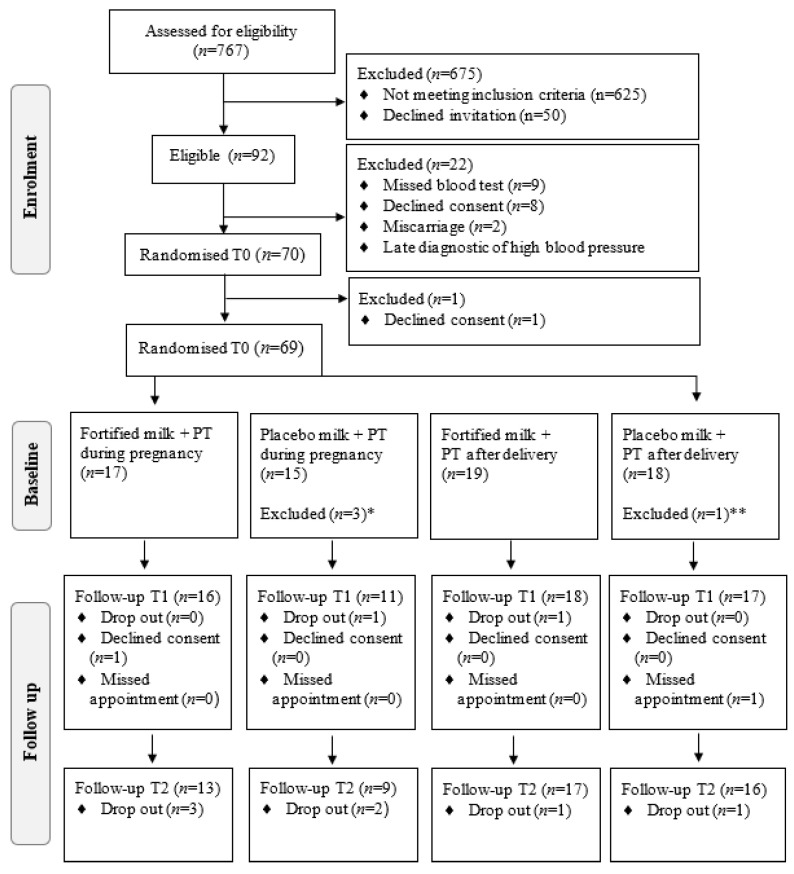
Flowchart of enrolment, allocation, and follow-up of the pregnant women from a low socioeconomic area in Rio de Janeiro. PT: Periodontal treatment. ^*^ Not started periodontal treatment (*n* = 1) or milk consumption (*n* = 1) or both (*n* = 1). ^**^ Not started milk consumption (*n* = 1).

The detailed study protocol for this 2 × 2 factorial randomised controlled feasibility trial with a parallel process evaluation i.e., the groups were not cross-over, has been described previously [27]. The reporting of this study is in accordance with the extension of the Consolidated Standards of Reporting Trials (CONSORT) statement for randomised pilot and feasibility trials [28]. Participants were allocated to one of four intervention groups using a permutated block randomisation matrix, stratified by smoking status (Figure 1) provided and concealed by Sealed Envelope Ltd. (London, UK). The intervention groups were as follows: 1 fortified sachet with vitamin D and calcium and powdered milk plus periodontal treatment (PT) during pregnancy, 2 placebo sachet and powdered milk plus PT during pregnancy, 3 fortified sachet and powdered milk plus PT after delivery and 4 placebo sachet and powdered milk plus PT after delivery. The study follows the CONSORT reporting standards.

### 2.2. Study Population and Eligibility Criteria

This study was conducted among adult (>18 years) pregnant women attending prenatal care at a Public Health Centre in Duque de Caxias in Rio de Janeiro, Brazil. During prenatal booking at the health centre, pregnant women were referred by nurses to the study team, who applied a preliminary eligibility checklist. To be eligible women had to be up to 20 weeks gestation at their first prenatal visit at the Health Centre, without a diagnosis of HIV/AIDS, syphilis, psychosis, diabetes prior to pregnancy, thyroid disease, any condition causing vitamin D hypersensitivity (e.g., lymphomatous disorders) or a history of renal stones or family history of renal stone and hyperparathyroidism. Participants reporting lactose intolerance and/or milk allergy, intake of antibiotics or any immune-suppressants or medication known to affect vitamin D/calcium metabolism, consumption of ≥4 servings/day of dairy products, or taking vitamin D supplements at >400 IU/day and using dental braces were excluded.

Those initially eligible were invited to take part in a dental screening, conducted by one trained dentist. Women with ≥1 tooth with at least one site with ≥4 mm of clinical attachment loss (CAL) and presence of bleeding on probing (BOP) on the same site were classified as having periodontitis and invited to take part in the study. Women with extensive dental cavities and decay were then excluded.

### 2.3. Intervention

The intervention included a serving of commercially available semi-skimmed powdered milk (20 g) and a sachet (2 g) with vitamin D_3_ (500 IU) and dairy calcium (CAPOLAC 500 mg) to be consumed twice a day during breakfast and afternoon snack or supper. Participants were advised to avoid milk consumption during main meals (lunch and dinner) to prevent concomitant intake of the prenatal iron supplements provided as part of the routine prenatal care in Brazil. Women in the control group were given the same amount of semi-skimmed milk powder with a non-fortified sachet (placebo) with similar colour, flavour, smell and texture. Participants were asked to reconstitute the 20 g of powdered milk and the content of the sachet into 200 mL of potable water for each serving. Sachets and milk were provided monthly to participants. Women were encouraged to drink the milk or add the content in preparations including porridges, smoothies, or kneaded fruits.

Right after randomisation, participants allocated to the early PT group underwent a conventional non-surgical PT throughout pregnancy up to childbirth, consisting of prophylactic dental polishing to remove the bacterial film, scaling and root planning, as necessary. The number of therapy sessions varied according to disease severity with a maximum of five sessions per participant. The treatment was performed by a trained dentist who was not involved in dental screening. Participants allocated to the late PT group started the treatment after childbirth as part of the routine dental care in the health centre. In each session, all women were given personalised oral hygiene instructions.

At the beginning of the study, all participants were given a brochure with information about the benefits of dairy food intake during pregnancy, a healthy diet and oral health hygiene instructions. Participants also received fluoride toothpaste, a toothbrush and dental floss. All women regardless of group allocation were advised not to change their routine physical activity neither consume any other supplements apart from the ones provided by the health centre (usually 400 μg/d folic acid and 60 mg/d ferrous sulphate). All women with young children (older than six months) were given a surplus of commercially available whole milk powder to ensure mothers did not share their milk provision with their families.

### 2.4. Data Collection

Baseline data (T0) were collected before randomisation and after checking for participant eligibility to the trial. Baseline data were collected up to the second trimester of pregnancy. The study included two additional follow-ups: T1 during the third trimester of pregnancy (30–38 gestational weeks) and T2 at 6–8 weeks postpartum.

At the three time-points (T0, T1 and T2), participants answered structured questionnaires about their general health, social support and lifestyle. Information on socioeconomic demographics was provided at baseline only.

Maternal blood samples were collected in the morning after 12-h overnight fast in all three-time points for analysis of biochemical markers. Blood samples were stored in a freezer with a temperature of −80 °C until analysis at the Institute of Nutrition Josué de Castro at the Federal University of Rio de Janeiro. Fasting serum glucose (mg/dL) levels were estimated by an enzymatic colorimetric method and fasting serum insulin (µU/mL) by chemiluminescence. Serum calcium concentrations (mg/dL) were analysed using an automated colorimetric technique, 25-hydroxyvitamin D (25(OH)D) serum concentrations (ng/mL) levels were determined by the method of high-pressure liquid chromatography (HPLC), serum ultra-sensitive C-Reactive Protein (CRP) (mg/L) levels were measured by the immunoturbidimetric method. Birthweight (g) information was extracted from the medical records.

### 2.5. Oral Health Examination

After the initial oral health screening during the recruitment phase, women with confirmed periodontitis who agreed to take part in the study underwent a full-mouth periodontal examination at baseline (T0) and postpartum (T2) regardless of group allocation. Periodontal examination was performed at six sites per tooth including mesiobuccal, mid-buccal, distobuccal, mesiolingual, mid-lingual and distolingual sites, using colour-coded North Carolina periodontal probes and a dental mirror. No x-ray was taken. Additionally, bi-monthly maintenance periodontal examinations were performed in those who had finished the PT during pregnancy. A tailored form was developed to register the data on gingival bleeding on probing (BOP), probing depth (PD), clinical attachment loss (CAL) and dental mobility. Calibrated and trained dentists performed oral examinations and PT. The dentists calibrated their probing force using a scale prior to all examinations to ensure that adequate pressure of approximately 20 g was applied [29]. The dentist who performed the periodontal examination was blinded regarding group allocation.

### 2.6. Analysis

The distribution of the outcomes was evaluated via visual inspections of charts and statistical tests for normality. The sample baseline characteristics were described by means and standard deviations (±SD) or absolute (*n*) and relative frequencies (%) in each time-point of each intervention group. One-way ANOVA and Pearson’s chi-squared tests were applied for comparison between intervention groups.

The results were presented using both intention-to-treat (ITT) and *per*-protocol analysis. The ITT refers to a comparison of the treatment groups that include all women as originally allocated after the randomisation. In the case of missing observations, the ‘last value carried forward’ approach was used [30]. The per-protocol analysis refers to the comparison of treatment groups that include only those participants who completed the treatment originally allocated [30].

The treatment variation over time (delta) across groups was mainly calculated as the difference between the mean values at the end of the study (T2) and baseline (T0) however mean differences in each time points (T1–T0 and T2–T1) were also estimated to aid interpretation of results as some parameters might have a different distribution during pregnancy and postpartum. Caution should be exercised when interpreting the delta values as the aim of this feasibility trial was not to assess effectiveness and therefore is underpowered to do this [28].

BOP is the main clinical outcome for this study. In addition to examining how BOP varied over time in different treatment groups, we are also interested in exploring potential interactions of effect modifiers on BOP beyond group allocation. For example, although serum calcium levels and insulin concentrations were initially considered in the analysis as metabolic outcomes, we also explored the independent impact of these variables on BOP. A factorial ANOVA type III that is robust for unbalanced factorial design and can identify any potential interactions between intervention groups was performed. However, it is important to consider that the study was not designed nor powered to identify statistically significant interactions and further ANOVA type II analyses were also used to explore the interventions interactions on all outcomes. For all statistically significant outcomes in the previous analyses, an analysis of covariance (ANCOVA) considering the remaining primary outcomes as influence factors was performed and results were graphically displayed. All tests considered an alpha of 0.05 with no corrections for multiplicity of tests. All analyses were performed using R statistical software version 4.0.2 (R Core Team, Vienna, Austria).

## 3. Results

The mean (±SD) age and gestational age of 69 women who participated in the study at the baseline was 27.9 (±5.7) years and 16.2 (±2.4) weeks, respectively. On average, women had 10.5 (±2.2) years of schooling and a pre-pregnancy BMI of 27.3 (±7.0) kg/m^2^. The majority of the women reported living with a partner (86.9%), self-ascribed as mixed or black skin colour (88.4%), did not smoke (88.4%) nor consumed alcohol during pregnancy (82.6%). The mean parity was 1.2 (1.2) and 65.2% had another child prior to the study enrolment. Regarding their oral health, the mean pocket depth was 4.2 (±0.2) mm. The clinical attachment loss was 4.3 (±0.2) mm and approximately one-quarter of the sites had bleeding on probing in all intervention groups at baseline. After randomisation, all baseline characteristics were equally distributed across intervention groups and no statistically significant differences were found between them (Table 1). Therefore, these potential confounders are unlikely to influence the impact of the intervention among groups.

Table 2 (*per*-protocol analysis) shows that the variation (delta: T2–T0) of BOP over time significantly differed between early and late PT groups. The mean BOP was significantly reduced in the early PT groups (Fortification: −0.1 ± 0.15; Placebo: −0.11 ± 0.15), while BOP increased (or worsened) in the late PT groups (Fortification: 0.04 ± 0.19; Placebo: 0.07 ± 0.15). There was a trend towards a slight reduction in both PD and CAL in the early PT compared to late PT, but results were not statistically significant (*p*-value > 0.05).

There was no significant difference in changes in calcium and vitamin D levels from T0 to T2 among intervention groups (Table 2). However, vitamin D levels tended to increase from T0 to T1 (pregnancy period) with the highest increase among the early PT plus Fortification group, but the results did not reach statistical significance at 0.05. Then, vitamin D levels fell after childbirth when the intervention ceased. No interactions between PT and fortification were detected on vitamin D and calcium levels.

Regarding the metabolic markers, insulin levels decreased over time across the intervention groups (Table 2). Although the late PT plus fortification group experienced the highest overall reduction (T2–T0) in insulin levels (−4.0 ± 2.7 µU/mL) compared to other groups, in the *post hoc* analysis with only the late PT group there was no significant difference between fortification and placebo. A detailed analysis (Table 3) revealed that there was a significant reduction in insulin levels from late pregnancy to postpartum (from T1 to T2) and this change was driven by the periodontal treatment, with those allocated in the late PT group experiencing the highest reduction in insulin levels. Therefore, the overall significant reduction in insulin levels among the late PT group was likely to be driven by changes from the 3rd trimester of pregnancy to 6–8 weeks postpartum. No significant differences in glucose levels were observed among the four intervention groups over time.

Regarding the inflammatory marker, CPR levels tended to reduce over time (delta: T2–T0) across the intervention groups; however, there was no significant difference between groups (Table 2). A detailed analysis Table 3) showed a more pronounced reduction in CRP during pregnancy (T1–T0, during pregnancy) in the early PT group compared to the late PT group but the *p*-value was borderline (0.09). On the other hand, there was a significant reduction in CRP levels from the 3rd trimester of pregnancy to the postpartum period in the late PT group compared to the early PT group.

In the ITT analysis, the results presented similar patterns as in the *per*-protocol (Supplementary Table S1).

**Figure 2:**
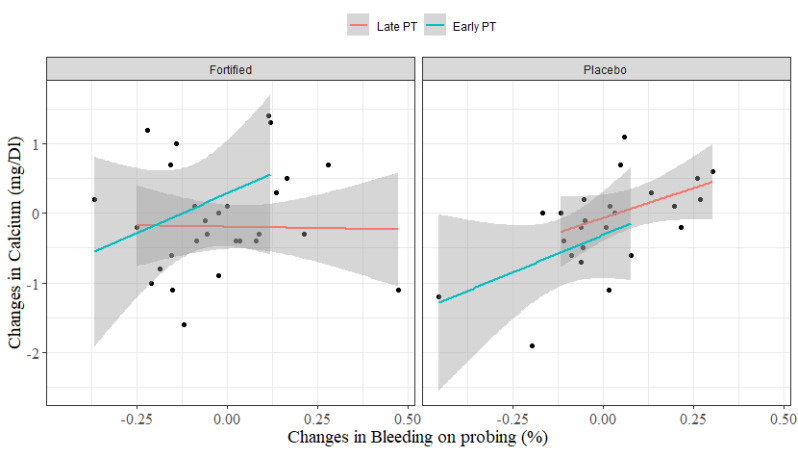
Difference in the periodontal treatment and milk fortification effects of interventions (delta) in calcium and bleeding on probing. **Late PT:** Periodontal treatment postpartum. **Early PT:** Periodontal treatment during pregnancy. **Fortified:** milk with the fortification of calcium and vitamin D. **Placebo:** milk without fortification. **Delta of calcium (mg/dL) and bleeding on probing (%):** levels in T2–levels in T0 (baseline).

The correlation of the main effect of the interventions between the outcomes by the ANCOVA analysis showed that BOP worsens as serum levels of calcium increase. However, this trend was not fully observed across all groups since the fortification and late PT group had the lesser pronounced trend, indicating some interaction effect between treatments (Sum Sq. = 2.07; *p*-value = 0.03) (Figure 2).

A delta equal to zero means no change between T0 and T2. Positive and negative results mean an increase and reduction in values from baseline, respectively. The results of ANCOVA test shows that the effect of bleeding on probing dental levels is significant at 10% in the delta levels of calcium (Sum of Squares = 1.51; *p*-value = 0.076) and the effect of the milk fortification combined with periodontal treatment is significant at 5% in the delta levels of calcium (Sum Sq. = 2.07; *p*-value= 0.03), all adjusted for the effect of milk fortification (Sum Sq. = 0.37, *p*-value = 0.37) and periodontal treatment (Sum Sq. = 1.07, *p*-value = 0.13, Sum Sq. Residual = 21.6). 

**Figure 3:**
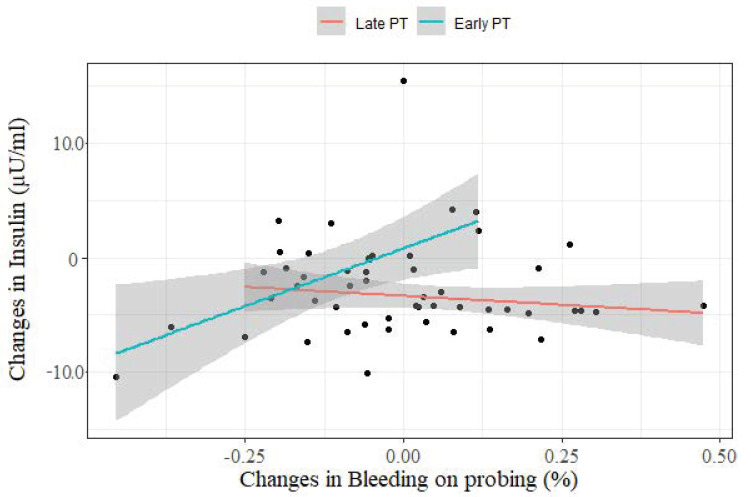
Difference in the effect of periodontal treatment intervention on delta insulin levels and delta bleeding on probing. **Late PT:** Periodontal treatment postpartum. **Early PT:** Periodontal treatment during pregnancy. **Delta of insulin and bleeding on probing:** levels in T2–levels in T0 (baseline).

Figure 3 shows that BOP was correlated with insulin levels indicating that if PT is not initiated during pregnancy the reduction expected in insulin levels overtime could have not been observed (Sum Sq. = 146.83; *p*-value ≤ 0.001). There was no difference between the fortification and the placebo group. Therefore, the analysis was not stratified by fortification group.

The results of ANCOVA test shows that the effect of periodontal treatment is significant at 5% in the delta levels of insulin (Sum of Squares = 210.03; *p*-value ≤ 0.001) and the impact of the periodontal treatment considering the delta levels of bleeding on probing is significative at 5% in the delta levels of insulin (Sum Sq. = 146.83; *p*-value ≤ 0.001), all adjusted for the effect of bleeding on probing alone (Sum Sq. = 8.49; *p*-value = 0.48; Sum Sq. Residual = 827.88).

## 4. Discussion

This exploratory analysis showed that periodontal treatment during pregnancy significantly decreased maternal BOP between baseline and postpartum even in a small sample of pregnant women. Periodontal disease is unpredictable and slowly progressive so that a clinically relevant difference in pocket depth and attachment loss would not be expected or detectable during the intervention period [31]. However, the absence of BOP is a patient observed health outcome and a reliable indicator of periods of periodontal disease stability and inflammation status [31]. A previous qualitative study about the oral health of pregnant women in public healthcare centres in Brazil showed that gingival bleeding was perceived as the most frequent problem arising from pregnancy [32]. However, most women reported that they did not seek dental care or discussed this problem with their physicians during routine prenatal care. The bleeding was considered a common pregnancy issue by the majority of women [32].

Periodontal disease is a prevalent condition during pregnancy. Studies show that the prevalence can vary from 30 to 70% [33]. Pre-existing gingivitis and periodontitis conditions are aggravated during the reproductive period due to hormonal changes [3,4,5]. High levels of circulating progesterone associated with placental growth and accompanied by the increased levels of oestrogens can lead to gingival capillary dilatation, bleeding, and inflammation [5].

While our results showed that PT during pregnancy improved BOP, there was a further tendency of a less pronounced increase in BOP among the late PT group receiving vitamin D/calcium-fortified milk, suggesting a potential protective impact of the fortification; however, results were not statistically significant. Although this finding is in line with previous literature, it is important to consider that our study was not powered to find a significant effect. A clinical trial with 85 Pakistani pregnant women (36 in the intervention group versus 49 in the placebo group) found that 4000 IU of vitamin D daily during 6 months of pregnancy (2nd and 3rd trimesters) did not result in significant improvements in BOP compared to the placebo group [34]. In that study, pregnant women did not receive PT [34]. Since vitamin D has immunomodulatory effects against bacterial infections it is expected that vitamin D in addition to PT might have a potential protective effect on periodontal clinical parameters.

Elevated CRP levels during gestation is a marker of inflammation [35]. As expected, there was an overall (T2–T0) reduction in CPR, which is a marker of inflammation, in both the per-protocol and the ITT analyses. We also observed a more pronounced reduction in the early PT compared to late PT during pregnancy (T1–T0) while women were undergoing PT treatment. In fact, women in the late PT and placebo group experienced an average increase of 1.2 mg/L in CRP levels during pregnancy. Although the direction of these results suggests a potentially beneficial effect, the results did not reach statistical significance. Additionally, a significantly higher reduction in CRP levels from the 3rd trimester of pregnancy to postpartum in the late PT group compared to the early PT group was observed. This higher decrease in CRP in the late PT group might be explained by the fact that levels of CRP increase during pregnancy above non-pregnant levels and then gradually reduces after childbirth [36]. The cause of such an increase is still unknown [36]. Women in the early PT, on the other hand, already experienced a reduction in CRP levels from the second trimester to the third trimester and therefore had a less pronounced further reduction in CRP levels from the third trimester to postpartum compared to women in the late PT.

The mean calcium levels tended to remain stable over time and there were no significant differences across groups despite the fortification. As all women consumed milk and therefore increased their overall calcium intake, this might explain why a significant difference in serum calcium levels between groups was not observed. The literature indicates that total serum calcium normally falls throughout pregnancy, particularly during the peak of foetal demand that occurs in the third trimester [37]. Thus, although the results did not reach statistical significance, this finding might indicate an overall protective effect of the intervention by adding milk to the diet on maternal calcium deficiency and long-term bone health. This finding highlights that a healthy and balanced maternal diet is critical to ensure that maternal nutritional needs, as well as the needs of the growing foetus, are met. In most low and middle-income countries, poor diets coupled with normal physiologic pregnancy changes can lead to micronutrient deficiency [38]. It worth noting that there are maternal adaptations during pregnancy to meet the additional calcium demands [37]. The intestinal calcium absorption is doubled as early as 12 weeks and this may allow women to store calcium in advance to provide for the peak foetal demand in the third trimester via calcium mobilization from the maternal skeleton [37]. Concomitantly, the 24-h urine calcium increases by 12 weeks gestation, which can exceed the normal range, indicating an increase in maternal intestinal calcium absorption [39].

In this study, vitamin D levels tended to increase from T0 to T1 and then reduce in T2 during the postpartum period. Contrary to expectation, we observed an overall (T2–T0) reduction in vitamin D levels in all groups. Although during the pregnancy period, vitamin D tended to be slightly higher in the fortification group compared to placebo in both early and late PT, results were not statistically significant. Also, we observed that vitamin D levels tended to reduce after childbirth when women were no longer consuming milk and undergoing periodontal treatment. However, previous studies have found that 25 (OH)D decreases during chronic periodontal inflammation while vitamin D concentration (25(OH)D) increases during acute periodontal inflammation periods because of increased 25-hydroxylase activity of periodontal cells [40]. We speculate that the same pattern might be observed in pregnant and postpartum.

Insulin concentrations tend to increase during pregnancy and decrease during postpartum [41]. Our results are consistent with the literature as we observed insulin reduction mainly in T2–T1, in all groups, and significantly in the fortified milk and placebo groups that received PT during the postpartum. This finding is compatible with hormonal changes that are expected in the postpartum period. Fasting insulin concentrations and insulin resistance increase during pregnancy, especially in the 2nd and 3rd trimester [41], as observed between T0 and T1 in this study. After delivery, insulin concentrations return to similar values to those of the pre-pregnancy period [41]. However, breastfeeding at least among obese women with gestational diabetes can influence postpartum insulin concentration. A previous study observed significant reduction in insulin resistance in obese women with gestational diabetes with high-intensity breastfeeding in the early postpartum phase [42]. In the present study, we did not register whether pregnant women breastfed or not, and could not examine if this influenced the results obtained in relation to the insulin concentrations in the postpartum period and the difference observed between groups. It is important to note, that even though there were some differences in insulin concentrations between groups, in all groups the mean insulin concentration was within the normal range proposed by Murguía-Romero et al. [43]. None of the participants developed gestational diabetes.

The fasting glucose concentrations tended to reduce during pregnancy (T0 to T1) and increase in the postpartum (T1 to T2) in all groups. These changes are consistent with previous literature [44]. Riskin-Mashiah et al. [44] observed a reduction of fasting glucose concentrations from the first to the third trimester and an increase after delivery. Our results suggest that PT and milk fortification potentially influenced glycaemic control during pregnancy and postpartum in women with periodontitis. However, it is important to highlight that all women consumed milk and a large prospective cohort study found that the intake of low-fat dairy was significantly and inversely associated with the risk of developing gestational diabetes [45]. The authors also reported that among pregnant women with a total dietary calcium intake below 1200 mg/day, a 200 mg per day increase in calcium intake was associated with a 22% reduction in the risk of gestational diabetes [45].

Our results also suggested that women allocated in the early PT plus fortification group gave birth to slightly heavier babies compared to the other groups. However, the results were not statistically significant. This finding might also be explained by the fact that all women consumed milk as part of the intervention. Milk consumption regardless of micronutrient fortification is associated with foetal growth [46,47]. This association seems to be driven by milk protein, or milk components closely associated with protein, rather than by the fat or carbohydrate fractions of milk [46,47]. We did not collect specific information on milk intake beyond what was supplied by the study. However, the study was conducted in a low-income area in Rio de Janeiro, Brazil and therefore milk consumption is expected to be low due to affordability issues. Therefore, a milk surplus was provided to prevent women from sharing their milk supply with their families and consequently ensure adequate consumption. In our previous study [24], we found an overall adherence to milk consumption (number of sachets consumed/ total number of sachets provided to participants) of 85.2%.

This exploratory study was designed to assess the feasibility of a multicomponent intervention delivered in a resource-poor setting and metropolitan area of Rio de Janeiro, Brazil. Therefore, a large sample size was not required. Thus, the main limitation of this study is the small sample size. Although inferential statistics were performed the main aim was to uncover any relevant and potential associations among the study variables.

Since we recruited a young population of women of childbearing age, it was anticipated that chronic severe periodontitis which tends to progress with age would not be prevalent. Thus, we applied less rigid diagnostic criteria defined as the presence of one or more teeth with at least one of the periodontal sites with ≥4 mm of clinical attachment loss with the presence of bleeding on probing on the same site. The presence of bleeding on probing on the same site ensured the existence of local inflammation. However, having applied stricter diagnostic criteria including ≥2 teeth with ≥4 mm of clinical attachment loss, only one pregnant woman would have been excluded from the study [25]. The rationale for recruiting women up to 20 weeks gestation was based on the previous literature suggesting that starting PT after the second trimester of gestation might be too late to reduce inflammation [48]. The literature also suggests that the number of PT appointments (e.g., 1 treatment course during 1–2 appointments) in previous RCTs might not have been effective in preventing the progression of periodontal disease during pregnancy, indicating that additional periodontal maintenance throughout gestation may be required [49]. Although previous studies concluded [48,49,50] that a pre-pregnancy treatment might be the best preventive measure of negative gestational outcomes associated with periodontitis, many pregnancies are unplanned and such trials would be very difficult to be conducted and would require a large sample size.

In conclusion, this study, although a feasibility exploratory clinical trial, has provided invaluable data to the emerging field of nutritional intervention for oral health as well as contributed to the literature on the importance of maternal oral health for systemic health and consequently gestational outcomes. A full-scale and definitive trial is needed to establish clinical effectiveness.

## Figures and Tables

**Table 1:** Baseline ^a^ characteristics by intervention groups of pregnant women with periodontitis from a low socioeconomic area in Rio de Janeiro, Brazil.

	Early PT		Late PT		
Variables	Fortification	Placebo	Fortification	Placebo	*p*-Value ^b^
Age (years) (mean, SD)	27.5 (6.4)	26.5 (6.3)	28.7 (6.0)	28.8 (4.3)	0.63
Schooling (years) (mean, SD)	10.2 (2.3)	10.5 (2.3)	10.7 (1.8)	10.7 (2.7)	0.93
Per-capita income (USD) ^c^ (mean, SD)	105.9 (60.5)	181.9 (146.6)	162.1 (111.5)	159.7 (106.6)	0.23
Marital status (*n*, %)					
Living with a partner	13 (21.6)	13 (21.6)	18 (30.0)	16 (26.6)	0.43
Other ^d^	4 (44.4)	2 (22.2)	1 (11.1)	2 (22.2)	
Self-reported skin colour (*n*, %)					
White	3 (37.5)	0 (0)	2 (25)	3 (37.5)	0.36 ^e^
Mixed or black	14 (22.9)	15 (24.5)	17 (27.8)	15 (24.5)	
Gestational age (weeks) (mean, SD)	16 (2.5)	15 (2.5)	17 (2.1)	16 (2.6)	0.60
Pre-pregnancy BMI (kg/m^2^) (mean, SD)	26.3 (6.5)	27.1 (8.0)	27.7 (4.5)	28.0 (9.2)	0.23
Parity (number of parturitions) (*n*, %)					
No previous delivery	7 (29.1)	5 (20.8)	7 (29.1)	5 (20.8)	0.86
One or more previous deliveries	10 (22.2)	10 (22.2)	12 (26.6)	13 (28.8)	
Smoking (*n*, %)					
No	16 (26.2)	13 (21.3)	16 (26.2)	16 (26.2)	0.85 ^e^
Current smoker	1 (12.5)	2 (25.0)	3 (37.5)	2 (25.0)	
Alcohol intake (*n*, %)					
No	15 (26.3)	12 (21)	15 (26.3)	15 (26.3)	0.90 ^e^
Yes	2 (16.6)	3 (25.0)	4 (33.3)	3 (25.0)	
Pocket depth (mm) (mean, SD)	4.3 (0.19)	4.21 (0.21)	4.22 (0.19)	4.28 (0.24)	0.51
Clinical attachment loss (mm) (mean, SD)	4.3 (0.20)	4.24 (0.23)	4.27 (0.19)	4.3 (0.27)	0.89
Sites with bleeding on probing (BOP) (mean, SD)	0.21 (0.15)	0.20 (0.14)	0.15 (0.11)	0.23 (0.16)	0.33

^a^ Baseline was between gestational weeks 11 and 21. ^b^ ANOVA test applied for continuous variables and Chi^2^ test for categorical variables. ^c^ Value originally measured in Brazilian Reais (BRL) but converted to USA dollars (USD). Exchange rate in February 2019, BRL 3.75 = USD 1. ^d^ Other = Not living with a partner or do not have a partner. ^e^ Fisher test applied. BMI, Body Mass Index.

**Table 2:** Main outcomes per-protocol according to intervention groups in pregnant and postpartum women participating in the IMPROVE trial.

	Early PT				Late PT				
	Fortification		Placebo		Fortification		Placebo		*p*
	*n*	Mean (SD)	*n*	Mean (SD)	*n*	Mean (SD)	*n*	Mean (SD)	Value
BOP (%)									
T0	13	0.23 (0.16)	9	0.21 (0.18)	17	0.15 (0.11)	16	0.23 (0.16)	
T2		0.13 (0.12)		0.09 (0.07)		0.19 (0.20)		0.29 (0.18)	
ΔT		−0.10 (0.15)		−0.11 (0.15)		0.04 (0.19)		0.07 (0.15)	**<0.01** (0.76)
PD (mm)									
T0	13	4.34 (0.20)	9	4.25 (0.24)	17	4.25 (0.19)	16	4.27 (0.25)	
T2		4.28 (0.19)		4.22 (0.26)		4.20 (0.13)		4.29 (0.17)	
ΔT		−0.06 (0.25)		−0.03 (0.19)		−0.05 (0.21)		0.02 (0.22)	0.66 (0.31)
CAL (mm)									
T0	13	4.34 (0.21)	9	4.32 (0.27)	17	4.29 (0.18)	16	4.29 (0.29)	
T2		4.32 (0.19)		4.29 (0.34)		4.37 (0.43)		4.29 (0.17)	
ΔT		−0.02 (0.23)		−0.03 (0.30)		0.08 (0.48)		0.00 (0.27)	0.18 (0.36)
Calcium (mg/dL)									
T0	13	9.0 (0.6)	9	9.3 (0.5)	17	9.3 (0.7)	15	9.0 (0.6)	
T1		8.7 (0.5)		8.8 (0.6)		8.9 (0.4)		9.0 (0.8)	
T2		9.1 (0.7)		8.7 (0.6)		9.2 (0.6)		9.0 (0.7)	
ΔT		0.1 (1.0)		−0.6 (0.7)		−0.1 (0.6)		0.1 (0.6)	0.53 (0.75)
Vitamin D (ng/mL)									
T0	13	25.2 (9.4)	9	30.1 (17.7)	17	30.5 (6.3)	16	31.5 (12.5)	
T1		29.9 (9.5)		27.3 (7.8)		31.2 (8.8)		32.6 (10.0)	
T2		23.4 (10.5)		28.8 (11.5)		27.2 (7.1)		28.1 (9.5)	
ΔT		−1.8 (9.5)		−1.3 (12.1)		−3.3 (6.7)		−3.5 (5.4)	0.43 (0.97)
CRP (mg/L)									
T0	13	12.3 (8.2)	9	8.9 (5.5)	17	11.2 (7.5)	16	8.8 (5.1)	
T1		8.3 (5.2)		6.3 (3.5)		12.7 (8.2)		10.8 (8.7)	
T2		6.4 (4.9)		7.6 (7.5)		7.2 (7.4)		4.6 (3.5)	
ΔT		−5.9 (7.1)		−1.3 (9.5)		−4.0 (7.8)		−4.2 (4.8)	0.90 (0.41)
Glucose (mg/dL)									
T0	13	71.3 (6.4)	9	73.4 (7.5)	17	73.9 (7.3)	16	72.7 (8.6)	
T1		77.7 (13.9)		68.0 (8.4)		73.7 (13.2)		78.8 (14.5)	
T2		80.2 (14.6)		75.9 (6.8)		78.5 (11.5)		81.3 (11.8)	
ΔT		8.9 (16.9)		2.5 (8.7)		4.6 (12.9)		8.6 (11.8)	0.92 (0.99)
Insulin (µU/mL)									
T0	13	7.7 (4.2)	9	7.2 (4.0)	17	9.0 (3.8)	16	7.5 (3.5)	
T1		8.9 (4.0)		9.0 (3.5)		11.4 (4.4)		10.3 (3.9)	
T2		7.6 (5.2)		5.7 (2.8)		5.0 (2.5)		4.5 (1.9)	
ΔT		−0.1 (7.2)		−1.5 (4.5)		−4.0 (2.7)		−3.0 (2.9)	**0.02** (0.95)
Birthweight (g)									
T2	13	3467.1 (327.0)	9	3266.8 (457.6)	17	3334.8 (385)	16	3404.8 (573.6)	0.85 (0.73)

Bold: *p*-value less than 0.05 for periodontal treatment (outside parenthesis) or milk fortification (inside parenthesis). BOP: Sites with bleeding on probing; CAL: clinical attachment loss; CRP: ultra-sensitive C-Reactive Protein; PD: pocket depth.

**Table 3:** Detailed analysis of changes in outcomes over time according to intervention groups in pregnant and postpartum women participating in the IMPROVE trial.

	Early PT		Late PT		
	Fortification	Placebo	Fortification	Placebo	*p*
	Mean (SD)	Mean (SD)	Mean (SD)	Mean (SD)	Value
Calcium (mg/dL)					
T1–T0	−0.36 (0.77)	−0.46 (0.85)	−0.38 (0.89)	0.06 (1.21)	0.37 (0.41)
T2–T1	0.42 (0.49)	−0.08 (0.64)	0.29 (0.71)	−0.04 (1.3)	0.81 (0.10)
ΔT	0.10 (1.0)	−0.60 (0.7)	−0.10 (0.6)	0.10 (0.5)	0.53 (0.75)
Vitamin D (ng/mL)					
T1–T0	3.98 (7.82)	−3.96 (15.6)	0.99 (7.53)	0.94 (8.3)	0.84 (0.17)
T2–T1	−6.49 (8.65)	1.59 (7.28)	−3.92 (7.71)	−3.52 (5.81)	0.69 (0.11)
ΔT	−1.80 (9.5)	−1.20 (12.1)	−3.30 (6.7)	−3.50 (5.4)	0.43 (0.97)
CRP (mg/L)					
T1–T0	−3.5 (7)	−3.2 (3.9)	−0.01 (12.3)	1.2 (8.6)	0.09 (0.72)
T2–T1	−1.9 (6.1)	1.3 (8.2)	−5.6 (10.6)	−5.9 (9.3)	**0.04** (0.67)
ΔT	−5.9 (7.1)	−1.3 (9.5)	−4.0 (7.8)	−4.2 (4.8)	0.90 (0.41)
Glucose (mg/dL)					
T1–T0	2.94 (14.4)	−3.40 (8.2)	0.22 (15)	3.20 (11.8)	0.67 (0.75)
T2–T1	2.58 (21)	7.90 (11.4)	4.80 (14.2)	2.70 (10.5)	0.79 (0.83)
ΔT	8.90 (16.9)	2.50 (8.7)	4.6 (12.9)	8.60 (11.8)	0.92 (0.99)
Insulin (µU/mL)					
T1–T0	0.3 (3.7)	1.3 (4.8)	2.4 (3.5)	1.7 (4.8)	0.21 (0.98)
T2–T1	−1.6 (6.5)	−3.3 (4.3)	−6.4 (3.6)	−5.6 (4.6)	**<0.01** (0.93)
ΔT	0.1 (7.2)	−1.5 (4.5)	−4.0 (2.7)	−3.0 (2.9)	**0.02** (0.95)

Bold: *p*-value less than 0.05 for periodontal treatment (outside parenthesis) or milk fortification (inside parenthesis).

## Data Availability

The data presented in this study are available on request from the corresponding author. The data are not publicly available due to ethical considerations, in accordance with the consent provided by participants on the use of confidential data.

## Supplementary Materials

The following are available online at https://www.mdpi.com/2072-6643/13/3/783/s1, Table S1: Main outcomes per intention to treat (ITT) analysis according to intervention groups in pregnant and postpartum women participating in the IMPROVE trial.
