# Supplementary material for: Exploratory Efficacy of Calcium-Vitamin D Milk Fortification and Periodontal Therapy on Maternal Oral Health and Metabolic and Inflammatory Profile
Source: Nutrients. 2021 Feb 27;13(3):783. doi: 10.3390/nu13030783 (PMC7997467; doi:10.3390/nu13030783)
Supplement: Supplementary file 1 [file nutrients-13-00783-s001.pdf]

Supplementary Material

Table S1. Main outcomes per intention to treat (ITT) analysis according to intervention groups in pregnant and postpartum women participating in the IMPROVE trial.

	Early PT				Late PT				
	Fortification		Placebo		Fortification		Placebo		*p-value*
	n	Mean (SD)	n	Mean (SD)	n	Mean (SD)	n	Mean (SD)	
**BOP** (%)									
T0	17	0.21 (0.15)	15	0.20 (0.14)	19	0.15 (0.11)	18	0.23 (0.16)	
T2		0.13 (0.12)		0.09 (0.07)		0.20 (0.20)		0.29 (0.18)	
*Δ*T		-0.10 (0.15)		-0.11 (0.15)		0.04 (0.19)		0.07 (0.15)	**<0.01** (0.76)
PD (mm)									
T0	17	4.30 (0.20)	15	4.24 (0.23)	19	4.27 (0.19)	18	4.30 (0.27)	
T2		4.27 (0.19)		4.22 (0.26)		4.20 (0.13)		4.29 (0.17)	
*Δ*T		-0.03 (0.24)		-0.02 (0.19)		-0.07 (0.21)		-0.01 (0.22)	0.76 (0.36)
CAL (mm)									
T0	17	4.3 (0.19)	15	4.21 (0.21)	19	4.22 (0.19)	18	4.28 (0.24)	
T2		4.30 (0.20)		4.29 (0.34)		4.37 (0.43)		4.29 (0.17)	
*Δ*T		0.00 (0.25)		0.07 (0.3)		0.13 (0.48)		0.01 (0.27)	0.22 (0.32)
**Calcium** (mg/dL)									
T0	17	9.1 (0.6)	14	9.2 (0.5)	19	9.3 (0.7)	18	9.0 (0.6)	
T1		8.8 (0.5)		8.7 (0.5)		8.9 (0.5)		9.0 (0.8)	
T2		9.1 (0.7)		8.7 (0.6)		9.2 (0.6)		9.0 (0.7)	
*Δ*T		0 (0.9)		-0.5 (0.6)		-0.1 (0.6)		0 (0.6)	0.25 (0.44)
**Vitamin D** (ng/ml)									
T0	17	26.6 (9.8)	15	28.1 (14.3)	19	30.6 (6.0)	18	31.5 (11.9)	
T1		29.4 (9.2)		25.8 (8.0)		31.6 (8.8)		33.1 (9.6)	

T2		23.4 (10.5)		28.8 (11.5)		27.2 (7.1)		28.1 (9.5)	
ΔT		-0.9 (8.5)		-2.7 (12.5)		-2.7 (6.8)		-2.4 (6.7)	0.68 (0.79)
**CRP** (mg/L)									
T0		11.1 (7.3)		9.8 (6.3)		11.9 (9.5)		8.7 (5.0)	
T1	17	7.8 (4.8)	15	5.3 (3.8)	19	12.5 (8.0)	18	10.1 (8.4)	
T2		6.4 (4.9)		7.6 (7.5)		7.2 (7.4)		4.6 (3.5)	
ΔT		-4.9 (6.4)		-2.1 (8.9)		-5.3 (9.4)		-4.1 (4.5)	0.57 (0.31)
**Glucose** (mg/dL)									
T0		72.8 (8.6)		74.0 (8.2)		73.5 (7.0)		73.6 (8.6)	
T1	17	76.5 (12.4)	15	68.8 (9.0)	19	74.0 (12.9)	18	77.2 (14.3)	
T2		80.2 (14.6)		75.9 (6.8)		78.5 (11.5)		81.3 (11.8)	
ΔT		4.9 (16.3)		3.0 (8.9)		4.7 (12.5)		5.9 (13.5)	0.71 (0.98)
**Insulin** (μU/ml)									
T0		7.9 (3.7)		9.8 (7.2)		8.7 (3.8)		7.9 (3.7)	
T1	17	8.4 (3.6)	15	8.8 (3.3)	19	11.4 (4.3)	18	10.0 (3.8)	
T2		7.6 (5.2)		5.7 (2.8)		5.0 (2.5)		4.5 (1.9)	
ΔT		-0.6 (6.4)		-1.4 (4.4)		-3.7 (3.0)		-3.0 (3.1)	**0.03** (0.09)
**Birthweight** (g)									
T2	13	3,467.1 (327.0)	9	3,266.8 (457.6)	17	3,334.8 (385)	16	3,404.8 (573.6)	0.85(0.73)

Bold: p-value less than 0.05 for periodontal treatment (outside parenthesis) or milk fortification (inside parenthesis).

BOP: Sites with bleeding on probing; CAL: clinical attachment loss; CRP: ultra-sensitive C-Reactive Protein; PD: pocket depth.
